# Genotoxicity of Heterocyclic PAHs in the Micronucleus Assay with the Fish Liver Cell Line RTL-W1

**DOI:** 10.1371/journal.pone.0085692

**Published:** 2014-01-09

**Authors:** Markus Brinkmann, Henning Blenkle, Helena Salowsky, Kerstin Bluhm, Sabrina Schiwy, Andreas Tiehm, Henner Hollert

**Affiliations:** 1 Department of Ecosystem Analysis, Institute for Environmental Research, RWTH Aachen University, Aachen, Germany; 2 Department of Environmental Biotechnology, Water Technology Center, Karlsruhe, Germany; Indian Institute of Toxicology Reserach, India

## Abstract

Heterocyclic aromatic hydrocarbons are, together with their un-substituted analogues, widely distributed throughout all environmental compartments. While fate and effects of homocyclic PAHs are well-understood, there are still data gaps concerning the ecotoxicology of heterocyclic PAHs: Only few publications are available investigating these substances using *in vitro* bioassays. Here, we present a study focusing on the identification and quantification of clastogenic and aneugenic effects in the micronucleus assay with the fish liver cell line RTL-W1 that was originally derived from rainbow trout (*Oncorhynchus mykiss*). Real concentrations of the test items after incubation without cells were determined to assess chemical losses due to, e.g., sorption or volatilization, by means of gas chromatography-mass spectrometry. We were able to show genotoxic effects for six compounds that have not been reported in vertebrate systems before. Out of the tested substances, 2,3-dimethylbenzofuran, benzothiophene, quinoline and 6-methylquinoline did not cause substantial induction of micronuclei in the cell line. Acridine caused the highest absolute induction. Carbazole, acridine and dibenzothiophene were the most potent substances compared with 4-nitroquinoline oxide, a well characterized genotoxicant with high potency used as standard. Dibenzofuran was positive in our investigation and tested negative before in a mammalian system. Chemical losses during incubation ranged from 29.3% (acridine) to 91.7% (benzofuran) and may be a confounding factor in studies without chemical analyses, leading to an underestimation of the real potency. The relative potency of the investigated substances was high compared with their un-substituted PAH analogues, only the latter being typically monitored as priority or indicator pollutants. Hetero-PAHs are widely distributed in the environment and even more mobile, e.g. in ground water, than homocyclic PAHs due to the higher water solubility. We conclude that this substance class poses a high risk to water quality and should be included in international monitoring programs.

## Introduction

One of the key processes that built the foundation of the organic chemical industry in Germany and many other European countries in the 19th century was the distillation of coal tar-oil, by which common building-blocks for numerous syntheses were derived, e.g. for textile dyes [1]. However, tar-oil and coal tar can contain up to 85% polycyclic aromatic hydrocarbons (PAHs) and 5–13% heterocyclic aromatic hydrocarbons containing nitrogen, oxygen or sulphur (hetero-PAHs) [2], [3], while the latter can constitute 40% of the water-soluble fraction. In industrial areas, e.g. gas plants, coke manufacturing or wood preservation sites, long ground water plumes contaminated with hetero-PAHs have been detected [4], [5], potentially also endangering drinking water resources [6] and aquatic ecosystems.

While PAHs were already extensively investigated with regard to fate, biodegradation, toxicology and ecotoxicology [7], [8], [9], only limited knowledge exists for the hetero-PAHs. Many studies focused on few individual compounds and toxicological effects, e.g. mutagenicity of carbazoles [10] or chromosome aberrations induced by quinolines [11]. Researchers just recently began to conduct comparative studies investigating a range of hetero-PAHs using different mechanism-specific *in vitro* bioassays. The biological effects so far cover, e.g. toxicity to *Daphnia* and green algae, mutagenicity in the Ames assay, embryotoxicity to embryos of the zebrafish (*Danio rerio*) or effects mediated by the aryl hydrocarbon and retinoid receptor, respectively [12], [13], [14], [15], [16], [17], [18].

To further fill the data gap mentioned above, we report on the first comparative study in which the clastogenic and aneugenic effects of heterocyclic PAHs typically found at tar-oil contaminated sites were investigated using the micronucleus assay with the permanent rainbow trout liver cell line RTL-W1 [19]. It is one of the best characterized cell lines derived from fish for mechanism-specific biotests [20] and was extensively used in genotoxicity studies [21], [22], [23], [24]. The *in vitro* micronucleus assay was validated [25] and standardized by an international guideline [26] with the Chinese hamster lung fibroblast cell line V79. Furthermore, it is well-accepted that a substance’s potential to induce micronuclei represents an important toxicological effect with potential adverse effects on the population level [21], [27], [28]. The substances tested were chosen to match the set of compounds identified earlier by the project framework KORA (retention and degradation processes to reduce contaminations in groundwater and soil) [12], [29] and comprised indole, 1-benzothiophene, benzofuran, 2-methyl benzofuran, 2,3-dimethyl benzofuran, quinoline, 6-methyl quinoline, carbazole, dibenzothiophene, dibenzofuran, acridine, and xanthene. To account for the dissipation of the compounds from the test medium, changes in chemical concentration during incubation were measured by means of gas chromatography-mass spectrometry (GC-MS) analyses [13].

## Materials and Methods

### Chemicals

Stock solutions of the used heterocyclic PAHs were prepared in dimethyl sulfoxide (DMSO). Indole (>99%), quinoline (>98%), carbazole (approx. 95%), 6-methylquinoline (>98%), benzothiophene (>98%), dibenzothiophene (>98%) were supplied by abcr (Karlsruhe, Germany). Acridine (>98%) was purchased from Merck (Darmstadt, Germany). Xanthene (99%), benzofuran (>99%), 2-methylbenzofuran (≥96%), 2,3-dimethylbenzofuran (≥97%) and dibenzofuran (approx. 98%) were supplied by Sigma-Aldrich (Deisenhofen, Germany).

### Micronucleus Assay with RTL-W1 Cells

The protocols recently published by Rocha et al. [30] for cell culture and the micronucleus assay with RTL-W1 cells were followed, with slight modifications. Cells were originally derived from rainbow trout liver [19] and generously provided by Drs Niels C Bols and Lucy Lee (University of Waterloo, Canada). Cells were cultured at 20°C in Leibovitz L15 medium with L-glutamine (Sigma–Aldrich) containing 9% fetal bovine serum (FBS, Biochrom, Berlin, Germany) and 1% (v/v) penicillin/streptomycin solution (Biochrom) according to Klee et al. [31]. Before use in the micronucleus assay, cells were rinsed twice with phosphate buffered saline (PBS, Sigma-Aldrich) and suspended following trypsinisation [32]. Cells of passage number 83 were used for the experiments.

**Figure 1:**
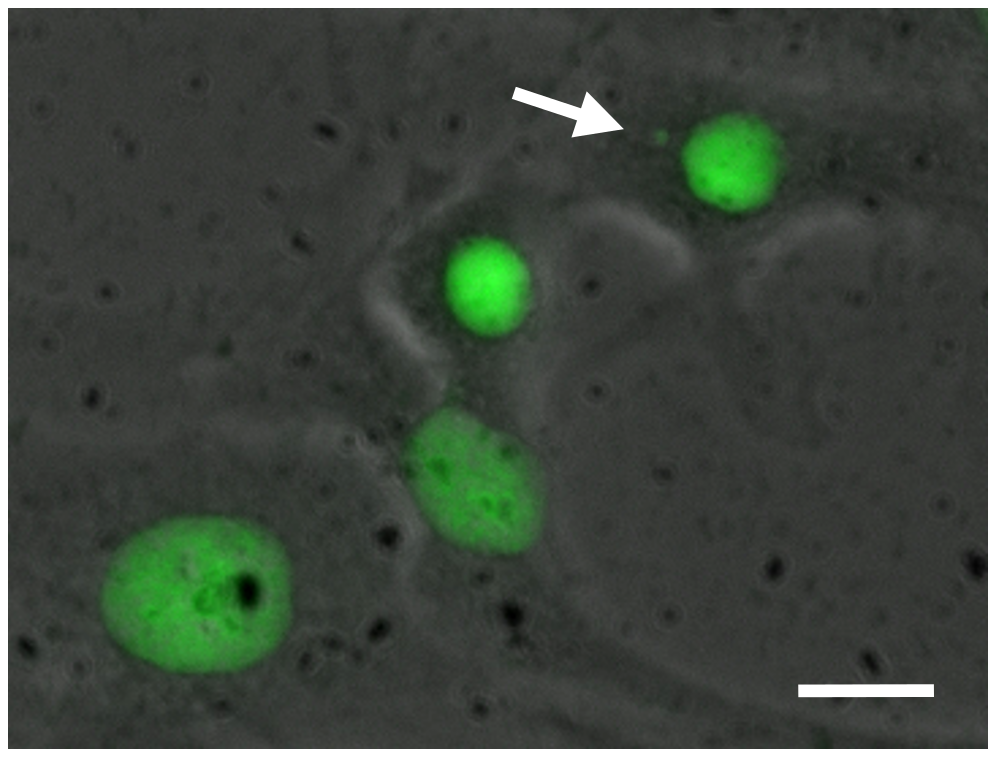
Composite photomicrograph of a micronucleus in RTL-W1 cells directly after cytokinesis (arrow). Nuclei and micronuclei were stained using Acridine Orange dye. The micrograph was captured at 1000× magnification and is a composite of bright-field and epifluorescence microscopy. Scale bar = 5 µm.

A volume of 2 ml of the cell suspension at a density of 5–6 10^4^ cells/ml was seeded onto ethanol pre-cleaned microscopic glass cover slips in 6-well plates (TPP, Trassadingen, Switzerland) and incubated for 12 h at 20°C (resulting in approx. 6–7 10^3^ cells/cm^2^) in a cooling incubator (Binder, Tuttlingen, Germany). Subsequently, the medium was aspired and completely exchanged with dilutions of the investigated hetero-PAHs and the plates incubated for 20 h at 20°C. For each substance, a serial dilution of each stock solution (1∶2) comprising five concentrations was tested in duplicate (i.e. on two different slides), while the highest concentration was equal to the NR_80_ of the substance, i.e. the concentration at which 80% viability of RTL-W1 cells was measured in the neutral red retention assay (Table 1). The maximum concentration of DMSO in the test was 1%. The exposure medium was aspired and completely exchanged with fresh L15 medium and the plates incubated for 72 h at 20°C to give cells enough time to divide at least once [33]. Subsequently, cells were fixed for 10 min in a PBS-diluted (1∶1 v/v) mixture of methanol and glacial acetic acid (4∶1 v/v). Fixation was repeated for 10 min in the undiluted mixture. After air-drying, the cover slips were mounted onto glass slides using DePeX (Serva, Heidelberg, Germany). Acridine orange was used for staining of the slides after fixation [34]. A total number of 2000 cells per slide were analyzed under an epifluorescence microscope (Nikon Instruments, Düsseldorf, Germany) with oil-immersion at 1000× magnification. The scoring criteria of the ISO guideline 21427-2 were used: (a) only cells with intact cellular structure were read, micronuclei shall have (b) the same staining intensity as and (c) a maximum size of about 30% of the main nucleus. Furthermore, cells must be (d) clearly separated from the nucleus [26]. A representative photomicrograph of a cell with micronucleus is shown in Figure 1.

**Table 1:** Corrected and uncorrected REPs relative to NQO, as well as respective corrected and uncorrected EC_25_ values in the micronucleus assay with RTL-W1 cells.

Substance	Maximum concentration^1^	Uncorrected EC_25_	Uncorrected REP	Corrected EC_25_	Corrected REP	Genotoxicity in mammalian models	LOQ	Loss
	mg L^−1^	mg L^−1^		mg L^−1^			µg L^−1^	%
4-nitroquinoline oxide (STD)	0.19	20.44 · 10^−3^	1					
Benzofuran	120.9	41.7	4.9 · 10^−4^	3.5	5.90 · 10^−3^	+ [51]	0.2	91.7
2-Methylbenzofuran	210.0	51.2	4.0 · 10^−4^	8.8	2.33 · 10^−3^	n.a.	0.2	82.9
2,3-Dimethylbenzofuran	110.0	n.d.	n.d.	n.d.	n.d.	n.a.	0.1	51.8
Dibenzofuran	56.9	21.8	9.4 · 10^−4^	5.0	4.12 · 10^−3^	− [52]	0.1	77.2
Benzothiophene	136.1	n.d.	n.d.	n.d.	n.d.	n.a.	0.2	75.0
Dibenzothiophene	40.8	10.8	1.9 · 10^−3^	3.2	6.45 · 10^−3^	n.a.	0.3	70.6
Acridine	40.9	10.3	2.0 · 10^−3^	7.3	2.82 · 10^−3^	+ [60], [61]	0.3	29.3
Xanthene	75.5	47.9	4.3 · 10^−4^	8.4	2.44 · 10^−3^	n.a.	0.1	82.5
Carbazole	25.6	7.5	2.7 · 10^−3^	3.5	5.81 · 10^−3^	+ [48]	0.2	53.1
Indole	162.7	55.5	3.7 · 10^−4^	11.6	1.76 · 10^−3^	n.a.	0.1	79.1
Quinoline	226.0	n.d.	n.d.	n.d.	n.d.	+ [11], [38], [39], [40], [41], [42], [43]	0.3	61.7
6-Methylquinoline	528.0	n.d.	n.d.	n.d.	n.d.	− [62]	0.3	41.1

Chemical losses in the micronucleus assay used for calculation of corrected EC_25_ (i.e. by multiplying the residual compound fraction with the EC_25_) and REP values were derived from GC-MS measurements in 6-well microplates without cells.

*n.d.: Inactive in assay system, i.e. substances which did not reach 25% induction of the NQO standard; n.a.: not available; STD: standard substance; LOQ: limit of quantification.*

^1^ *Maximun tested concentrations based on cytotoxicity data from Hinger & Brinkmann et al.* [*13*].

### Calculation of EC and REP Values

Induction factors (fold-changes) relative to blanks, i.e. controls without treatment containing only the Leibovitz L15 medium, were calculated for each concentration by dividing the micronucleus rate of the respective concentration level by the mean of the blank replicates. Resulting concentration-response curves for individual substances and the well-characterized standard substance NQO (4-nitroquinoline oxide) were calculated with the software GraphPad Prism 5 (GraphPad, San Diego, CA, USA) using the four-parameter logistic equation model following log-transformation of the concentration values. Since a number of samples did not exceed 50% effect of the maximum NQO concentration, fixed-effect-level based REP (relative potency) values (Equation 1) were calculated according to Brack et al. [35]. Unlike in receptor-mediated assays, these REP values cannot be used for mass-balance analyses (i.e. to answer which portion of a measured effect is caused by which compound classes or single compounds) due to the different possible modes of action and are just intended for reference and comparison among samples.
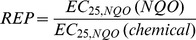
(1)


Effect concentrations (EC_25,NQO_) refer to the concentration of the substance causing 25% of the maximum effect level of NQO. The arbitrary level of 25% effect was chosen since it includes more valid curves than using the EC_50_ but is still within the linear portion of the concentration-response curves. The EC_25_ value for NQO, EC_25,NQO_ (NQO), was derived from the same test repetition of the micronucleus assay.

### Chemical Analysis

To account for the effects of e.g. volatilization or sorption to plastic plates, real concentrations were compared to nominal concentrations comparable to the methodology recently published in Hinger & Brinkmann et al. [13] and Peddinghaus et al. [17]. Here, 6-well microplates were prepared in the same way as for the micronucleus assay without adding cells. Before addition to the plate, and after a 20 h incubation period in the microplate, solutions were stored in a glass vial with PTFE cap. The heterocyclic PAHs were extracted (liquid – liquid extraction) with methyl *tert*-butyl ether (MTBE). For this purpose 45 mL of the diluted sample were spiked with 10 µL internal standard solution in acetone (0.63 µg/µl toluene d_8_ and 0.66 µg/µl naphthalene d_8_, both obtained from Merck, Darmstadt, Germany) and extracted with 5 mL MTBE. Extraction time was 20 minutes. After phase separation, the extract was dried with sodium sulphate and subsequently analysed using gas chromatography (Agilent technologies GC 6890 N). The GC was equipped with an autosampler (Agilent technologies) and mass selective detector (MSD) (Agilent technologies MS 5973 Network) operated in SIM (Selective Ion Monitoring) mode. For Separation of the substances, a ZB-5 Inferno column (60 m×0.25 mm×0.25 µm) by Phenomenex was used. The concentrations of the internal standard in samples and external standards were equal. The limit of detection was 0.1 to 0.3 µg L^−1^ for the investigated substances. To correct the bioassay data for the measured chemical losses, EC_25_ values were multiplied with the remaining compound fraction and the REPs re-calculated with the corrected EC_25_.

### Statistical Analysis

All spreadsheet calculations were performed using Microsoft Excel™ 2007. Statistical analyses were conducted with Sigma Stat 3.11 (Systat Software, Erkrath, Germany). All tested treatments and levels were tested for statistically significant differences from the blanks, i.e. controls without treatment, by use of one-way ANOVA (*p*≤0.001). Dunnett’s test was used as the multiple range test to identify significant differences between treatments and blanks. The probability of Type I error (α) was set to *p*≤0.05. Values are expressed as mean value ± standard deviation, unless indicated.

## Results and Discussion

### Clastogenic and Aneugenic Effects of Heterocyclic PAHs

**Figure 2:**
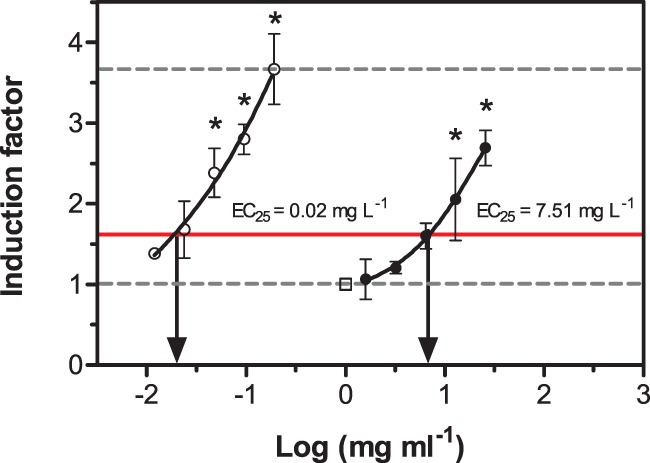
Exemplary concentration-response curve (carbazole) measured in the micronucleus assay with RTL-W1 cells (closed circles). The concentration-response curve for NQO (open circles) and the blanks, i.e. control cells without treatment (open square and lower dashed line), are given for reference. Induction factors are fold-changes relative to blanks. EC_25_ values relative to the maximum induction of NQO (upper dashed line) were calculated to derive fixed-effect-level-based REPs. Concentration values on the x-axis refer to nominal medium concentrations of the substances. Circles represent mean values measured in duplicate experiments, error bars the standard deviation. The red line depicts 25% of the maximum induction caused by the standard NQO. Asterisks denote statistically significant differences compared to blanks (one-way ANOVA with Dunnet’s test, *p*≤0.05).

**Figure 3:**
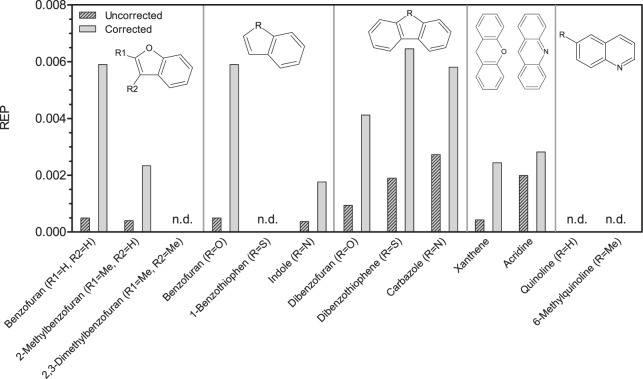
Uncorrected and corrected relative potency (REP) values of hetero-PAHs. Uncorrected values refer to nominal concentrations and corrected to measured concentrations in the exposure medium after incubation without cells. *N.d.: inactive in assay system, i.e. substances which did not reach the stipulated level of 25% induction of the NQO standard.*

**Figure 4:**
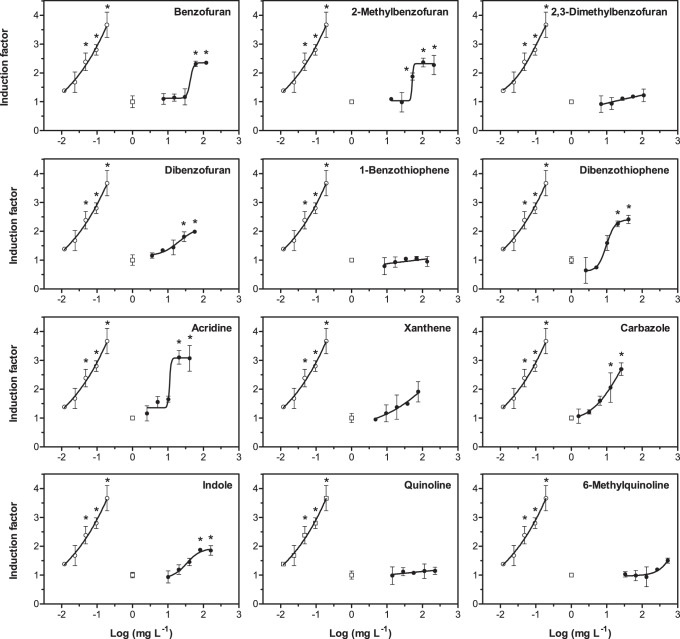
Dose-response curves in the micronucleus assay with RTL-W1 cells for all investigated heterocyclic compounds (closed circles). Standard curves for NQO (open circles) and blanks, i.e. control cells without treatment (open squares, grey line), are given for reference. Induction factors are fold-changes relative to blanks. Concentration values on the x-axis refer to nominal medium concentrations of the substances. Dots represent mean values measured in duplicate experiments, error bars the standard deviation. Asterisks denote statistically significant differences compared to blanks (one-way ANOVA with Dunnet’s test, *p*≤0.05).

The substances 2,3-dimethylbenzofuran, benzothiophene, quinoline and 6-methylquinoline did not cause significant induction of micronuclei in the permanent fish liver cell line RTL-W1, i.e. did not reach 25% induction of the nitroquinoline oxide (NQO) standard (Table 1). Acridine caused the highest absolute induction (approx. 3-fold compared to blanks). Carbazole, acridine and dibenzothiophene were the most potent substances. To be able to better compare the investigated substances, relative potency (REP) values were calculated, comparing the potency of the reference compound NQO with the potency of the test item. Figure 2 illustrates this data analysis approach. REP values (Figure 3) ranged from 3.7 · 10^−4^ (indole) to 2.7 · 10^−3^ (carbazole). The three substances with the highest potency were only approx. 500-fold less potent than 4-nitroquinoline oxide. Full concentration-response curves are given for reference (Figure 4). While concentrations of hetero-PAHs in contaminated aquifers are typically in the µg L^−1^ range, values up to the mg L^−1^ range (at which effects in the micronucleus assay were observed) have occasionally been detected at highly contaminated sites [29]. However, care should be taken when comparing *in vitro* results with aqueous exposure concentrations, since substances with differing physicochemical properties can be absorbed and accumulated to a varying extent and through different tissues and organs [36].

Eisenträger et al. [12] found that out of the tested substances (similar to the set tested here) only quinoline, 6-methylquinoline and xanthene caused mutagenic effects in the Ames assay with *Salmonella typhimurium,* while these effects were only apparent in treatments with metabolic activation, i.e. when supplemented with rat liver S9. The same effects for the 6-methylated and the parent quinoline were found by Debnath et al. [37]. The latter substance was also shown to cause significant induction of liver micronuclei and chromosome aberrations in rats and the hamster lung fibroblast cell line CHL/IU [11], [38], [39], [40] and is a potent hepatocarcinogen in mice and rats [41], [42], [43]. Indole was positive in the Ames assay [44]. Acridine was shown to be positive in the SOS chromotest with *Escherichia coli* K12 and a yeast-based reporter-gene assay in which DNA damage induces the expression of green fluorescent protein (GFP) via the RAD54 promoter [45]. It was not mutagenic in the Ames assay [12], [46] but produced clastogenic effects detected with the yeast DEL assay without metabolic activation [47]. Carbazole was demonstrated to be clastogenic by Jha et al. [48] but was not mutagenic in the Ames assay [49] or carcinogenic in mice [43]. The O-heterocyclic compound benzofuran showed very interesting effects, although being negative in the Ames assay [12], [50]; DNA damage, as measured with the Comet assay, as well as the formation of micronuclei was higher in female specimens, both *in vivo* in rat kidney and *in vitro* using primary rat and human kidney cultures [51]. Dibenzofuran, a substance that was previously reported not to be genotoxic in a mammalian system was identified to be potent genotixicant in the fish system [52].

The following general trends can be deduced: For heterocyclic three-ring PAHs with two benzene rings fused with one five-membered aromatic ring (dibenzofuran, dibenzothiophene, carbazole), the genotoxic potential decreased in the following order of hetero-atoms: nitrogen>sulphur>oxygen. A comparable trend was observed for the three-ring hetero-PAHs with central six-membered ring, where the toxicity of acridine (nitrogen hetero-atom) was markedly higher than that of xanthene (oxygen hetero-atom). In accordance with the aforementioned studies, the azaarenes had the highest potency to induce genotoxic effects. Furthermore, for derivates of benzofuran, methylation had a decreasing effect on the genotoxicity (benzofuran >2-methylbenzofuran >2,3-dimethlybenzofuran).

Interestingly, the molar effect concentrations observed in the present study significantly correlated (Pearson’s correlation coefficient *r* = 0.88, *p* = 0.02, data not shown) with the embryotoxic potential of hetero-PAHs as observed by Peddinghaus et al. [17]. In this study, acridine and carbazole caused the highest toxicity to embryos of *Danio rerio* and showed the highest potency to induce micronuclei in RTL-W1 cells in the present study. Furthermore, Hinger & Brinkmann et al. [13] have demonstrated that these two substances had the highest relative potency for luciferase induction in the DR-CALUX assay, an assay for aryl hydrocarbon receptor (AhR) agonists, and generally seem to be of high concern. The metabolic pathways of PAHs and hetero-PAHs were lately reviewed by Xue et al. [53] and need to be considered when investigating the genotoxic effects of these substances.

### Influence of Substance Losses during Incubation

Chemical losses during incubation ranged from 29.3% (acridine) to 91.7% (benzofuran) and may lead to a drastic underestimation of the real potency of the substances, e.g. in mass-balance analyses (Table 1). Here, we attempted to correct for chemical losses from the exposure medium by correcting EC_25_ and REP values by applying analytically-derived correction factors. These correction factors were based on the compound concentration after incubation for 20 h (without cells but under the same conditions) and represent a worst-case scenario in which only the residual concentration after incubation caused the effect. The resulting REP values differed by a factor of 1.5–2 for substances with low losses during the incubation period (acridine and carbazole) compared to the uncorrected REP values. For benzofuran, the substance with the highest loss (91.7%), the REPs differed by a factor of 12 compared with the uncorrected value (Figure 3). As it has been shown by other studies from the KORA project framework, accounting for the chemical losses is a prerequisite to adequately judge the real toxicological potency [12], [13], [17]. Furthermore, experimental precautions are warranted when experimenting with these volatile genotoxicants.

### Implications for Water Quality

The substances we investigated here showed a relatively high potency to induce micronuclei in the permanent cell line RTL-W1. We were able to show genotoxic effects for six compounds that have not been reported for vertebrate systems before. Dibenzofuran was positive in our investigation and tested negative before in a mammalian system. Homocyclic PAHs are commonly monitored as priority pollutants and are indicator substances for contamination with substances originating from historically contaminated sites, e.g. gas-plants, wood preservation or dyestuff industry. However, heterocyclic aromatic compounds are markedly more mobile than homocyclic PAHs, e.g. in ground water, due to higher water solubility. Because ground water wells are an important drinking water resource, we conclude that this substance class poses a high risk to human health and should be included in groundwater monitoring programs, e.g. according to the German LAWA-GFS (threshold values for groundwater) that are based on toxicological and ecotoxicological effect data [54] or the health-related indicator value (HRIV) introduced by the German Federal Environment Agency (UBA) [55]. The international NORMAN network, which was originally founded by the European Commission to set up a permanent network of reference laboratories and research groups, recently emphasized the importance of also screening for compounds that are not commonly part of monitoring programs [56]. In fluvial environments, heterocyclic PAHs contribute to the overall genotoxicity. In studies applying the concept of effect-directed analysis (EDA) to sediment and suspended particulate matter samples, as well as soil samples from related flood plains, those fractions containing heterocyclic substances showed strong mutagenic or genotoxic effects [57], [58]. Re-suspension of contaminated sediments can ultimately lead to an increased bioavailability of such particle-bound pollutants with potentially adverse effects in aquatic biota [59].
